# Role of biomarkers in the management of antibiotic therapy: an expert panel review: I – currently available biomarkers for clinical use in acute infections

**DOI:** 10.1186/2110-5820-3-22

**Published:** 2013-07-09

**Authors:** Anne-Marie Dupuy, François Philippart, Yves Péan, Sigismond Lasocki, Pierre-Emmanuel Charles, Martin Chalumeau, Yann-Eric Claessens, Jean-Pierre Quenot, Christele Gras-Le Guen, Stéphanie Ruiz, Charles-Edouard Luyt, Nicolas Roche, Jean-Paul Stahl, Jean-Pierre Bedos, Jérôme Pugin, Rémy Gauzit, Benoit Misset, Christian Brun-Buisson

**Affiliations:** 1 Département de Biochimie, Hopital Lapeyronie CHU Montpellier, France, 371, avenue du doyen Gaston Giraud, 34295 Montpellier Cédex 5, France; 2 Service de Réanimation polyvalente, Groupe hospitalier Paris Saint Joseph, 185 rue Raymond Losserand, 75014 Paris, France; 3 Laboratoire de Microbiologie, Institut Mutualiste Montsouris, 42, Bld Jourdan, 75014 Paris, France; 4 Pôle d’Anesthésie Réanimation, CHU d’Angers, 4 rue Larrey, 49933 Angers Cedex 9, Angers, France; 5 Service de réanimation médicale, CHU Dijon, Université de Bourgogne, 14 rue Paul Gaffarel, 21970 Dijon, France; 6 Laboratoire Interactions Muqueuses Agents Pathogènes, EA562, UFR Médecine, Université de Bourgogne, 7 Bd Jeanne d’Arc, 21000 Dijon, France; 7 Service de Pédiatrie Générale, CHU Necker Enfants Malades, AP-HP & Université Paris Descartes, 149 rue de Sèvres, 75743 Paris, France; 8 Inserm, U953 Paris, France; 9 Département d’Urgences Médicales, Centre Hospitalier Princesse Grace, 1 avenue Pasteur, BP 489, 98012 Principauté de, Monaco; 10 Centre d’investigation clinique (INSERM CIE 1), 7 Boulevard Jeanne d’Arc, 21079 Dijon, France; 11 Clinique Médicale et Service d’Urgences Pédiatriques, Hôpital Mère-Enfant, CHU Nantes, 38 boulevard Jean-Monnet, 44093 Nantes cedex 1, France; 12 Pôle d’Anesthésie-Réanimation, Hôpital de Rangueil, CHU de Toulouse, 1, Ave Pr Jean Poulhès, TSA 50032, 31059 Toulouse Cedex 9, France; 13 Service de Réanimation Médicale, Institut de Cardiologie, Groupe Hospitalier Pitié-Salpêtrière, AP-HP & Université Pierre et Marie Curie - Paris VI, 4783,– boulevard de l’Hôpital, 75651 Paris Cedex 13, France; 14 Service de Pneumologie et Soins Intensifs Respiratoires, Hôpitaux Universitaires Paris Centre, AP-HP & Université Paris-Descartes, 27 rue du fbg St Jacques, 75679 Paris, France; 15 Service de maladies infectieuses et tropicales, Université 1 de Grenoble, CHU de Grenoble, BP 217, Boulevard de la Chantourne, 38043 Grenoble, France; 16 Service de réanimation, Centre hospitalier de Versailles, 177, rue de Versailles, 78150 Le Chesnay, France; 17 Intensive Care - SIRS Unit, University Hospitals of Geneva, 4 rue Gabrielle Perret-Gentil, 1211 Geneva 14, Switzerland; 18 Unité de réanimation, CHU Hôtel Dieu, AP-HP, Place du Parvis-de-Notre-Dame, 75004 Paris, France; 19 Centre de Recherche Clinique, Groupe hospitalier Paris Saint Joseph & Université Paris Descartes, 75014 Paris, France; 20 Service de Réanimation médicale, Hôpitaux Universitaires Henri Mondor, AP-HP & Université Paris-Est, 94000 Créteil, France; 21 Inserm U957, Institut Pasteur, Paris, France

**Keywords:** Infection, Sepsis, Emergency medicine, Biomarkers, Procalcitonin, C-reactive protein, sTREM-1, suPAR, proADM, Presepsin

## Abstract

In the context of worldwide increasing antimicrobial resistance, good antimicrobial prescribing in more needed than ever; unfortunately, information available to clinicians often are insufficient to rely on. Biomarkers might provide help for decision-making and improve antibiotic management. The purpose of this expert panel review was to examine currently available literature on the potential role of biomarkers to improve antimicrobial prescribing, by answering three questions: 1) Which are the biomarkers available for this purpose?; 2) What is their potential role in the initiation of antibiotic therapy?; and 3) What is their role in the decision to stop antibiotic therapy? To answer these questions, studies reviewed were limited to recent clinical studies (<15 years), involving a substantial number of patients (>50) and restricted to controlled trials and meta-analyses for answering questions 2 and 3. With regard to the first question concerning routinely available biomarkers, which might be useful for antibiotic management of acute infections, these are currently limited to C-reactive protein (CRP) and procalcitonin (PCT). Other promising biomarkers that may prove useful in the near future but need to undergo more extensive clinical testing include sTREM-1, suPAR, ProADM, and Presepsin. New approaches to biomarkers of infections include point-of-care testing and genomics.

## Review

### Introduction

Good antibiotic prescribing-which often means less prescribing-is of major concern to physicians nowadays, both because of high levels of antibiotic consumption in hospitals, and of the increasing prevalence of antimicrobial resistance, even if rates of methicillin-resistant *Staphylococcus aureus* have decreased recently in many European countries since the early 2000s. The principal objective of antibiotic prescribing is to ensure appropriate therapy when needed, while avoiding unnecessary or unduly prolonged therapy. Within this framework, obtaining adequate microbiological information is of paramount importance; unfortunately, such information is lacking in more than 50% of clinical situations where antibiotic therapy is prescribed, even in hospitalized patients. Whereas clinical information is usually sufficient to initiate empiric therapy, they lack accuracy to tailor subsequent therapy and decide on its duration. Physicians’ decisions would be strengthened if they could get help from results of accurate biomarkers reflecting the diagnosis or evolution of the infectious processes. The field of infection-associated biomarkers has grown rapidly within the past few years and is still expanding; few of them, however, have gone through the hurdles of rigorous testing in the clinical arena to allow specifying their role in clinical practice.

An 18-member expert panel convened under the auspices of the Maurice Rapin Institute, a not-for-profit independent physicians’ association (http://www.institutmauricerapin.org), to provide a state-of-the-art assessment of the currently available biomarkers and their potential role as an aid to the management of antibiotic therapy for acute infections. This report is a summary of their work and conclusions.

To frame the appraisal of the potential clinical role of biomarkers, the panel was asked to answer three formatted questions, as follows:

1. Which are the currently available biomarkers of the host’s response, those that are routinely available and which may contribute to the management of antibiotics in acute infections, and what are the limitations to the interpretation of their results in this context?

2. What is the potential contribution of such biomarkers to the initial decision of antibiotic prescription, and does this vary according to the characteristics of infection (i.e., site of infection, comorbidities, mode of acquisition, severity of presentation)?

3. When can biomarkers help make decisions to stop antibiotic therapy, and which factors mitigate their clinical use in this process?

The panel discussion was based on an analysis of the available literature through December 2012, after making the a priori decision to limit publications considered for answering questions 2 and 3 to clinical studies fulfilling the following criteria:

Having enrolled a minimum of a substantial number of patients (i.e., >50 patients);

Performed within less than 15 years (i.e., published since 2000);

Pertaining to biomarkers available for routine testing in hospitals’ laboratories.

The first part of this paper deals with the first question asked to the panel, and the second part deals with questions 2 and 3.

## Currently available biomarkers of the host

### Definition and role of a host’s biomarker

Biomarkers from the host can be anatomical, physiological, biochemical (either circulating or membrane-bound), or molecular markers. The latter two categories are detected within a tissue or biological fluid (e.g., blood, cerebrospinal fluid, or urine) and their presence or absence, or over- vs. under-expression is the judgment criteria. Of note, more than 90% currently available biomarkers are used only within research program and have not been introduced within the field of clinical biology.

#### Definitions

Currently accepted definitions for biomarkers have emerged from an expert panel driven by the U.S. National Institute of Health [1] and from regulatory definitions issued by the European Medicines Agency. A biomarker is “a biological characteristic, objectively measured (i.e., with acceptable accuracy and reproducibility) and used as an indicator for a physiological or pathological process, or of the activity of a medicine.” According to the NIH panel [1,2], biomarkers can be stratified in two categories (Table 1): *prognostic markers*, allowing to stratify patients according to their individual risk of having a specified outcome, independently of therapy (or of the lack of therapy), and *predictive markers*, which allow to predict the potential benefit (efficacy) and/or the risks (toxicity) of a therapy according to the biomarker status (absent/present).

**Table 1:** **Definition of biomarkers and subtypes according to the national institute of health**[1]

**Denomination**	**Definition**
Biomarker	Biological characteristics objectively measured, and used as a marker either of a normal or pathological biological pathway, or of a pharmacological response to a specific intervention
Biomarker type 0	Biological maker of the disease course, linked to a recognised clinical variable
Biomarker type I	Biological marker reflecting the effects of a therapy, and linked to its mechanism of action
Biomarker type II	Biological marker used as a surrogate endpoint, where changes in the biomarker levels are associated to a clinical benefit or to an increased risk.

In clinical practice, two types of biomarkers can be identified, which follow different development and validation pathways:

Those used independently from a specific therapy, as a diagnostic test, or for follow-up or prognosis, which will only be discussed in this paper from the viewpoint of infectious processes;

Those used as a companion to treatment, to select patients who may benefit from a specific therapy or used during follow-up of therapy as early predictors of efficacy or of treatment toxicity.

#### The ideal biomarker in infectious diseases

Within the field of infectious diseases, a biomarker may be used for identifying a high risk group or predisposing condition, as an aid to identification of the disease, or to direct therapy and stratify patients according to their specific risk factors, and/or as an aid to therapeutic management in order to avoid relapse of infection. An ideal biomarker for infection would combine diagnostic, prognostic, and follow-up of therapy characteristics and should be easily and rapidly available for routine clinical use (Table 2).

**Table 2:** **Important characteristics of biomarkers for clinical use in acute infections** (**from**[3])

**Criteria for use**	**Characteristics**
Diagnostic test	General: known preanalytic and analytic (accuracy, reproducibility) as well as physiological (intra and interindividual) variability, integrated in the interpretation of assay results
	High predictive values
	Ability to differentiate sepsis and noninfectious SIRS (specificity)
	Ability to differentiate acute viral from bacterial infection
Prognostic test	Early detection of patients at risk of a complicated course
	Levels associated with the inflammatory response (i.e., correlated to the severity of presentation and/or to organ dysfunctions)
	Predictor of mortality
Therapeutic test	Follow-up of the efficacy of a therapy (e.g., rapid kinetics, independent of organ dysfunction)
Accessibility	Routinely available
	Good acceptability to patients (i.e., noninvasive)
	Rapid turnaround time
	Easily interpreted
	Low cost

#### Potential role of biomarkers in acute infections: performance measurements

Biomarkers are expected to provide an assessment of the severity of infection or predict a complicated course to help making a decision on the best therapeutic approach and appropriate site of care (i.e., hospital or ambulatory care, intensive or ward care). Foremost, they should help the physician to decide about introducing or maintaining antibiotic therapy.

Within the recent years, dozens of potential biomarkers of infection have been described, and their analysis is a complex task. Current trends are to use a combination of biomarkers—notably cytokines—with multiplex tests providing simultaneous measurements of several biomarkers from a single biological sample. The major point is to examine whether their clinical performance and utility can be transposed to acute care situations.

The diagnostic performance of biomarkers is usually measured in terms of sensitivity (probability of a positive test among affected patients), specificity (probability of a negative test in unaffected patients), and by likelihood ratios and area under the ROC (*Receiver Operating Characteristics*) curves. Ideally, a biomarker would be both highly sensitive and specific; however, very sensitive tests provide few false-negative results, whereas highly specific ones provide few false-positive results. In emergency medicine practice, more emphasis is usually put on sensitivity (and negative predictive value, NPV), as the primary objective is to rule out the disease, whereas specificity (or positive predictive value, PPV) is emphasized when the objective is to confirm a clinical diagnosis. For quantitative tests, establishing ROC curves allows to select the best compromise between sensitivity and specificity of the test, according to which approach is emphasized. When a low threshold for positivity of the test is selected, its sensitivity increases but its specificity is lowered.

Sensitivity and specificity are however defined within a population where the patients’ status (“infected” or “noninfected”) is known, which does not corresponds to the population seen by the physician in his routine clinical practice. The clinical utility of a biomarker is therefore best assessed by measuring its predictive values (both positive and negative, PPV and NPV) and changes between pre- and post-test likelihood ratios in a given clinical context.

Two important points, often overlooked in the literature, should be considered when assessing the operating characteristics of biomarkers:

The characteristics of the population studied and of the “control group” (i.e., noninfected). For example, it is quite different to analyse a group of patients with a systemic inflammatory response (SIRS) following cardiac surgery (where the severity and prevalence of infection is low) or patients with SIRS within the context of pancreatitis evolving since >1 week, and both the severity and prevalence of infection are higher, with a high clinical impact of diagnosing infected pancreatitis necrosis.

Criteria used as the “gold standard” for defining infection (or lack thereof) [4,5].

### Limitations to the interpretation of biomarker levels

Improved measurement methods have largely enhanced the potential for biomarkers to identify patients at high risk of death or a complicated course, whether individual patients or the general population. Nevertheless, persisting difficulties arise when interpreting measurements of biomarker levels, a problem that is compounded by the dissemination of multiplex tests [6], thus increasing the volume of information generated. For some biomarkers, a threshold value can be determined, which allows a simple binary interpretation, but inevitably results in loss of precision; however, this approach cannot be generalised.

Interpreting biomarker levels can be problematic because of the variability of measurements resulting from several factors:

A lack of standardisation between different methods,

Biological factors, including preanalytical variables (tubes and transport media, time from sampling to analysis, etc.), analytical (precision, reproducibility, threshold of measurement, etc.), and intra- or interindividual variations; such factors must be assessed and controlled for before providing an interpretation of assays results.

In addition, prudent interpretation is mandatory when the known sensitivity or specificity of the biomarker measured is <90% or when the number of subjects studied is small. Moreover, in many studies, a single point in time has been obtained for biomarker measurement, and the lack of repeated measurements does not allow the use of such marker for adapting the duration of therapy.

We conclude that standardisation of measurement methods and guideline for the interpretation of biomarker levels in acute infections is mandatory before introducing their measurements into clinical practice. This development phase, including the determination of associated quality criteria (i.e., reproducibility and variation coefficient, threshold for detection), identification of confounding factors and corrective factors must be investigated. Finally, medico-economic evaluation is usually lacking and should be performed before proposing their introduction into routine clinical use.

### Biomarkers currently available for optimising antibiotic therapy

More than a hundred biomarkers have been studied in the serum of septic patients [7-9]. Few of them however are eligible for entering the clinical arena (see Additional file 1: Table S1) and being used for optimising antibiotic therapy because of limitations to the interpretation of results from these studies. Assays used often are not standardised (especially for ELISA and “multiplex” tests), making it difficult to compare results from different studies. Some techniques are difficult to adapt to the emergency context (multiplex tests, ELISA or high-flux cytometry). Some biomarkers cannot be presently retained because of a poor performance, of studies limited to a small population (e.g., <50 patients) or too scarce to allow conclusions on their potential utility. A limited number of biomarkers are currently of established or potential clinical interest within the field of acute infection.

#### Routinely available biomarkers

Two biomarkers fulfill the selection criteria mentioned above and are routinely available: C-Reactive protein (CRP) and procalcitonin (PCT). CRP has been tested in various conditions, but only a few of these studies have focused on its use for optimising antibiotic therapy. A single, prospective, randomized, controlled trial performed in the 1990s in children is available [10]; other studies have compared an intervention group to historical controls [11,12]. Despite the few available studies confirming its usefulness, CRP measurements are widely used in children to adjust the duration of therapy. Several studies are ongoing, testing the usefulness of CRP measurements as an aid to shorten the duration of therapy in adult patients having sepsis, community-acquired pneumonia or exacerbation of chronic obstructive pulmonary disease (COPD). Pending results from these studies, the use of CRP cannot be recommended at present as an aid to the initiation or discontinuation of antibiotics in adults; in children, however, CRP can probably be used to help discontinuing therapy, although the evidence is limited.

Procalcitonin has been more widely tested for optimising antibiotic therapy in both children and adults. In adults presenting with community-acquired lower respiratory tract infections (LRTI), several randomized, controlled trials (RCTs) have tested the use of PCT as an aid to the initiation and/or discontinuation of antibiotics and have been summarised in a recent individual patient meta-analysis [13-17]. Four of these studies enrolled more than 900 patients hospitalised in intensive care or high-dependency units [18-21]. Two well-designed studies have been performed in children: one study included 121 neonates having early sepsis [22] and another studied 384 children aged 1 to 36 months with acute fever of undetermined origin (Manzano, Bailey et al. 2010; Esposito, Tagliabue et al. 2011).

In view of these studies, the inclusion of PCT measurements within decision algorithms of antibiotic management for specific infections is likely appropriate (refer to Part II). However, further studies are needed in infections which have been insufficiently examined so far (i.e., most infections other than LRTI) to better define the role of PCT in the antibiotic strategy.

#### Recent biomarkers of potential interest in the near future

Intensive efforts are being made in the search of new diagnostic and prognostic biomarkers, which may be helpful for the management of antibiotic therapy in acute infections. In adults, four of these, the soluble Triggering Receptor Expressed on Myeloid cells-1 (sTREM-1), Soluble urokinase-type Plasminogen receptor (suPAR), proadrenomedullin (ProADM), and Presepsin appear promising. These four biomarkers are of reasonably easy access, have demonstrated acceptable sensitivity and/or specificity, and have been studied in a substantial number of patients to merit further consideration in adults. In children or neonates, too few and heterogeneous studies have been conducted with these new biomarkers to allow recommending any of these for potential introduction in the clinical arena at the present time; further studies are needed in these age groups.

##### sTREM-1

A member of the immunoglobulin superfamily, TREM-1 is a surface receptor of mature polymorphonuclear and monocytes cells contributing to innate immunity. Its expression is up-regulated when phagocytic cells are exposed to bacterial and fungal pathogens, but not during other non-septic inflammatory processes. TREM-1 amplifies the inflammatory response by increasing the production of pro-inflammatory cytokines while inhibiting IL-10 synthesis. During up-regulation of the surface receptor TREM-1, the soluble form sTREM-1 increases in biological fluids (blood, broncho-alveolar lavage fluid, CSF), where it can be assayed by ELISA using commercial immunoassay kits.

Several clinical studies [23-29] have tested the diagnostic and prognostic value of sTREM-1 (Table 3). Measurements in samples taken at the site of infection (CSF, BAL, pleural fluid) appear of higher clinical significance than plasma measurements.

**Table 3:** **Clinical experience with the use of sTREM**-**1 in acute infections**

**sTREM**-**1**	**References**	**Syndrome/ disease studied**	**Sampling**
Diagnostic value	[23]	Pneumonia	Plasma
	[24]	Pneumonia	BAL
	[25]	Meningitis	CSF
	[26]	Meningitis	CSF
Prognostic value	[27]	SIRS, sepsis, severe sepsis, septic shock	Plasma
	[28]	Sepsis, severe sepsis, septic shock	Plasma
	[29]	Sepsis, septic shock	Plasma

##### suPAR

suPAR (soluble urokinase-type plasminogen activator receptor) or CD87 is a widespread receptor for inflammatory response. Its constitutive expression is limited to some cell types, such as endothelium and leucocytes (polymorphonuclear, monocytes/macrophages). Its gene expression is under control of immune and inflammatory effectors, such as bacterial products (LPS), cytokines (IFN-gamma, TNF-alpha, IL-1-beta), and growth factors (FGF-2, VEGF, TGF-beta, EGF). During the inflammatory and immune response, the expression of suPAR is up-regulated on epithelial cells, leucocytes (lymphocytes), smooth muscle cells and fibroblasts; it also is up-regulated during tumour growth and metastatic tumour dissemination. Measurements can be obtained from commercial ELISA kits; suPAR measurements also are included in multiplex assays together with cytokines.

suPAR is of limited value as a diagnostic test. Its clinical value appears associated with its ability to identify patients at risk (Table 4) and might be of interest for the management of HIV patients receiving antiretroviral therapy [30], during the follow-up of patients who have nonpulmonary mycobacterial infection [31] and in children who have *Plasmodium falciparum* malaria [32]. suPAR also might be useful for the management of antibiotics in patients with sepsis [33-35], but this approach needs more extensive evaluation.

**Table 4:** Clinical experience with the use of suPAR in acute infections

**suPAR**			
**Clinical value**	**References**	**Syndrome/disease**	**Sampling**
Diagnostic value	[33,34]	Sepsis	Plasma
Pronostic value	[33-35]	Sepsis	Plasma

##### Pro-ADM

Adrenomedullin (ADM) is a 52-amino acids peptide, and a marker of the *CALC* gene family, acting as a mediator of cell proliferation, hormone regulation and embryogenesis. ADM is produced by endothelial cells, where it induces vasodilatation and maintains homeostasis. Pro-hormone fragments (pro-ADM) are more stable than the complete peptide and their levels can be measured in biological fluids by automated methods using the TRACE (Time-Resolved Amplified Cryptate Emission) method after immuno-capture. ProADM secretion increases during the immune response to viral or bacterial products in relation to the importance of the stimulation.

Pro-ADM is a biomarker of prognostic value (Table 5). Added to a clinical pneumonia severity score [36], pro-ADM could be used to identify the more severe patients for close monitoring and/or needing ICU care [37-40].

**Table 5:** Clinical experience with the use of pro-ADM in acute infections

**proADM**			
**Clinical value**	**References**	**Syndrome/disease**	**Sampling**
Diagnostic value	-	-	
Prognostic value	[38-40]	Pneumonia	Plasma

##### Presepsin

Presepsin (formerly CD14), is a glycoprotein receptor occurring at the surface of monocytes/macrophages. CD14 binds to lipopolysaccharide (LPS) complexes and LPS binding protein (LPB), which triggers the activation of toll-like receptor 4 (TLR4), resulting in the production of numerous pro-inflammatory cytokines. Following Presepsin activation by bacterial products, the CD14 complex is released in the circulation as its soluble form (sCD14), which in turn is cleaved by a plasma protease to generate a sCD14 fragment called sCD14-subtype (sCD14-ST). Plasma levels of sCD14 can be measured using an automated chemo-luminescent assay (PATHFAST®, Ingen®, France).

The most recent of the 4 biomarkers analysed, presepsin is both sensitive and specific and might be helpful to differentiate SIRS from sepsis associated with a bacterial infection [41-43] (Table 6).

**Table 6:** Clinical experience with the use of Presepsin in acute infections

**Presepsin**			
**Clinical value**	**References**	**Syndrome/disease**	**Sampling**
Diagnostic value	[41,42]	SIRS, Sepsis	plasma
Pronostic value	[43]	SIRS, Sepsis, Severe sepsis	plasma

We conclude that information gathered so far on these four biomarkers— sTREM-1, suPAR, proADM, and presepsin—suggest that they may have a role in future clinical developments, whether as diagnostic tests, or for stratification of patients by type of insult or severity, or to assess the therapeutic activity and efficacy and during follow-up of patients. To date, there are too few studies of the impact of these new biomarkers on the antibiotic management of patients and larger studies are required in this field.

### Future developments

Micro-RNAs (miR) are recently discovered potential candidate biomarkers. miR are small molecules (about 20 nucleotides) present in eucaryotic cells, which act as biologic regulators by modulating posttranscriptional regulation. They are ubiquitous and abound in the lung, liver, and kidney. After binding the corresponding smRNA sequence, they regulate gene expression by a repressor effect or by altering its target. A mi-RNA can bind to several smRNA. Their expression can be measured by RT-PCR and quantitative PCR.

Their multiple potential roles in positive or negative regulation of gene expression have been uncovered since the early 2000s, and dysfunctions of miR expression have been implicated in numerous human diseases (http://www.miR2Disease.org/), such as various types of cancers (“oncomir”), cardiomyopathy, or central nervous system diseases. miR also have been implicated in defense mechanisms against viral infections, where they may contribute to controlling viral infections. Integrated in the viral genome, a number of miR can regulate viral mRNA such as Epstein-Barr, cytomegalovirus, herpes, hepatitis C virus as well as the host’s RNA. Among bacterial infections, a role for miR has been suggested in *M*. *tuberculosis* infections by modulating the monocytes/macrophages interactions with the bacterium or regulating the expression of resistance gene or virulence factors. Modulation of the inflammatory response to infection with *H*. *pylori* also has been attributed to miR [44], notably miR-155 [45].

The spectrum of miRNAs initially released in blood and leucocytes of patients with septic shock differs from that of control patients. The three most dysregulated miR are miR-150, miR-182, miR-342-5p; miR-150 interferes with the development of an immune response by lymphocytes and thus might be a potential candidate as an early diagnostic and/or prognostic marker [46].

Other miRNAs have been associated with a high probability of a poor outcome in patients with septic shock: miR-223, miR-15a, miR-16, miR-122, miR-193*, and miR-483-5p. Based on individual AUROC for each miR, prediction of death varied between 0.61 (95% confidence interval (CI) 0.523-0.697) and 0.79 (95% CI 0.719-0.861) but reached 0.953 (95% CI 0.923-0.983) when combining the seven parameters [47].

Thus, miR might be potential candidates as early diagnostic and/or prognostic markers in sepsis. Numerous studies are needed with these new markers to better understand their role in biochemical and immunobiology processes in humans before their use for diagnostic and stratification of patients, prognostication, or therapeutic decision can be considered.

Two main technological advances are in progress, including 1) the development of point-of-care testing, with the availability of miniaturised and portable machines, allowing rapid testing at the bedside, even for sophisticated measurements (e.g., flux cytometry), which have been confined to specialised laboratories up to recently; and 2) the development of new methods, including the analysis of gene expression (genomics), of ARN activation (transcriptome), of production of proteins (proteomics), of lipids (lipidomics), or of metabolites (metabolomics). It is likely that these progresses will allow identifying new markers for better identification of patients, stratification of prognosis, and targeting therapy.

## Abbreviations

ADM and pro-ADM: Adrenomedullin and pro-adrenomedullin; aPTT: Activated partial thromboplastin time; AUROC: Area under the receiver operating curve; BAL: Broncho-alveolar lavage; BM: Bacterial meningitis; CAP: Community-acquired pneumonia; CCR3: Chemokine (C-C motif) receptor 3; CRP: C-Reactive protein; CRTH2: Chemoattractant receptor-homologous molecule expressed on Th2; CSF: Cerebrospinal fluid; DNI: Differential count of immature PMN; ELISA: Enzyme-linked immuno-sorbent assay; G-CSF: Granulocyte colony-stimulating factor; HLA: Human leukocyte antigens; HMGB1: High mobility group protein B1; ICAM 1: Intercellular adhesion molecule 1; ICU: Intensive care unit; IFN-γ: Interferon-gamma; IL: Interleukin; IP-10: Interferon gamma-induced protein 10; LBP: Lipopolysaccharide binding protein; LPS: Lipopolysaccharide; LRTI: Lower respiratory tract infection; MC: Monocytes; MCP-1: Monocyte chemotactic protein-1; MIF: Macrophage migration inhibitory factor; MR-proADM: Mid-regional proadrenomedullin; NIH: U.S. National institute of health; NPV: Negative predictive value; PAI 1: Plasminogen activator inhibitor 1; PCT: Procalcitonin; PMN: Polymorphonuclear neutrophil; PPV: Positive predictive value; ProADM: Proadrenomedullin; ProANP: Proatrial natriuretic peptide; ROC: Receiver operating characteristic curve; ROS: Reactive oxygen species; SAA: Serum amyloid A protein; sCD14-ST: Soluble CD14 subtype; sELAM: Soluble endothelial leucocyte adhesion molecule-1; sFlt-1: Soluble fms-like tyrosine kinase-1 or sVEGFR1; sPLA2: Soluble phospholipase A2; sTREM-1: Soluble triggering receptor expressed on myeloid cells-1; suPAR: Soluble urokinase-type plasminogen activator receptor; sVEGFR1: Vascular endothelial growth factor receptor 1 soluble; TNF: Tumor necrosis factor; TLR-2 or 4: Toll-like receptor 2 or 4; TRACE: Time-resolved amplified cryptate emission; uMIF: Urinary macrophage migration inhibitory factor; uMIF/cr: uMIF/Creatinine; VCAM-1: Vascular cell adhesion molecule 1; VEGF: Vascular endothelial growth factor.

## Competing interests

Meetings of the expert panel group were supported in part by an unrestricted grant from Thermo-Fisher to the Maurice Rapin Institute. This company had no role in the work of the panel, or in the drafting, revision or final approval of the manuscript.

A-MD declared participation as an investigator to the UTAPE study, sponsored by Thermo-Fisher, and her institution received funding for analytic studies of CT-proAVP using Kryptor.

CB-B was an investigator in the Prorata trial.

MC holds a patent for the MENINGITEST in Europe (European patent EP1977244), USA and Canada, and has a patent pending for the REFLUTEST (WO2010/109089).

C-EL was an investigator for the Prorata trial, and received lectures honoraria from Thermo-Fisher and Biomérieux.

NR is coordinating the UTAPE study on biomarkers in COPD exacerbations seen in the emergency department, sponsored by Thermo-Fischer.

P-EC received speaking fees from Thermo-Fisher.

Y-EC received research funding from Thermo-Fisher, Roche Diagnosis and Nephrotek.

CG-LG received funding by Thermo-Fisher for attending a scientific meeting.

JP received speaking fees for giving lectures within symposia organized by ThermoFischer.

J-PB, P-EC, RG, SL, BM, J-PQ, FP, YP, and J-PS have declared no competing interest in relation to the subject of this manuscript.

## Authors’ contributions

All panel members contributed to the panel discussions and analyses. Each panel members contributed to drafting different sections of the manuscript: A-MD, YP, FP, SR, and BM drafted part I; J-PQ, SL, Y-EC, J-PS, CG-L, MC, and RG drafted part II; and C-EL, NR, J-PB, JP, and CB-B drafted part III. A-MD, J-PQ, C-EL, RG, BM, MC, and CB-B extensively reviewed the consolidated manuscript and all authors approved its final version.

## Supplementary Material

Additional file 1: Table S1 List of biomarkers tested in the field of infectious diseases. Click here for file

## References

[B1] Atkinson AJ Colburn WA DeGruttola VG DeMets DL Downing GJ Hoth DF Oates JA Peck CC Schooley RT Spilker BA Biomarkers and surrogate endpoints: preferred definitions and conceptual framework Clin Pharmacol Ther 2001 3 89 95 11240971 10.1067/mcp.2001.113989

[B2] De Gruttola VG Clax P DeMets DL Downing GJ Ellenberg SS Friedman L Gail MH Prentice R Wittes J Zeger SL Considerations in the evaluation of surrogate endpoints in clinical trials. Summary of a National Institutes of Health workshop Control Clin Trials 2001 3 485 502 10.1016/S0197-2456(01)00153-2 11578783

[B3] Barraud D Gibot S CNERM Offenstadt G Apport des Marqueurs Biologiques de l’Infection aux Urgences et en Réanimation Réanimation Médicale 2009 2 Paris: Masson 988 992

[B4] Kaplan JM Wong HR Biomarker discovery and development in pediatric critical care medicine Pediatr Crit Care Med 2011 3 165 173 10.1097/PCC.0b013e3181e28876 20473243 PMC2924462

[B5] Schuetz P Christ-Crain M Muller B Procalcitonin and other biomarkers to improve assessment and antibiotic stewardship in infections–hope for hype? Swiss Med Wkly 2009 3 318 326 19529989 10.4414/smw.2009.12584

[B6] Dupuy AM Kuster N Lizard G Ragot K Lehmann S Gallix B Cristol JP Performance evaluation of human cytokines profiles obtained by various multiplexed-based technologies underlines a need for standardization Clin Chem Lab Med 2013 1 9 23314551 10.1515/cclm-2012-0648

[B7] Lever A Mackenzie I Sepsis: definition, epidemiology, and diagnosis BMJ (Clinical research ed) 2007 3 879 883 10.1136/bmj.39346.495880.AE PMC2043413 17962288

[B8] Pierrakos C Vincent JL Sepsis biomarkers: a review Crit Care 2010 3 R15 10.1186/cc8872 20144219 PMC2875530

[B9] Standage SW Wong HR Biomarkers for pediatric sepsis and septic shock Expert Rev Anti Infect Ther 2011 3 71 79 10.1586/eri.10.154 21171879 PMC3033193

[B10] Ehl S Gering B Bartmann P Hogel J Pohlandt F C-reactive protein is a useful marker for guiding duration of antibiotic therapy in suspected neonatal bacterial infection Pediatrics 1997 3 216 221 10.1542/peds.99.2.216 9024449

[B11] Bomela HN Ballot DE Cory BJ Cooper PA Use of C-reactive protein to guide duration of empiric antibiotic therapy in suspected early neonatal sepsis Pediatr Infect Dis J 2000 3 531 535 10.1097/00006454-200006000-00008 10877168

[B12] Jaswal RS Kaushal RK Goel A Pathania K Role of C-reactive protein in deciding duration of antibiotic therapy in neonatal septicemia Indian Pediatr 2003 3 880 883 14530549

[B13] Christ-Crain M Stolz D Bingisser R Muller C Miedinger D Huber PR Zimmerli W Harbarth S Tamm M Muller B Procalcitonin guidance of antibiotic therapy in community-acquired pneumonia: a randomized trial Am J Respir Crit Care Med 2006 3 84 93 10.1164/rccm.200512-1922OC 16603606

[B14] Briel M Schuetz P Mueller B Young J Schild U Nusbaumer C Periat P Bucher HC Christ-Crain M Procalcitonin-guided antibiotic use vs a standard approach for acute respiratory tract infections in primary care Arch Intern Med 2008 3 2000 2007 discussion 2007–2008 10.1001/archinte.168.18.2000 18852401

[B15] Schuetz P Christ-Crain M Thomann R Falconnier C Wolbers M Widmer I Neidert S Fricker T Blum C Schild U Effect of procalcitonin-based guidelines vs standard guidelines on antibiotic use in lower respiratory tract infections: the ProHOSP randomized controlled trial JAMA 2009 3 1059 1066 10.1001/jama.2009.1297 19738090

[B16] Burkhardt O Ewig S Haagen U Giersdorf S Hartmann O Wegscheider K Hummers-Pradier E Welte T Procalcitonin guidance and reduction of antibiotic use in acute respiratory tract infection Eur Respir J 2010 3 601 607 10.1183/09031936.00163309 20185423

[B17] Schuetz P Briel M Christ-Crain M Stolz D Bouadma L Wolff M Luyt CE Chastre J Tubach F Kristoffersen KB Procalcitonin to guide initiation and duration of antibiotic treatment in acute respiratory infections: an individual patient data meta-analysis Clin Infect Dis 2012 3 651 662 10.1093/cid/cis464 22573847 PMC3412690

[B18] Nobre V Harbarth S Graf JD Rohner P Pugin J Use of procalcitonin to shorten antibiotic treatment duration in septic patients: a randomized trial Am J Respir Crit Care Med 2008 3 498 505 10.1164/rccm.200708-1238OC 18096708

[B19] Hochreiter M Kohler T Schweiger AM Keck FS Bein B von Spiegel T Schroeder S Procalcitonin to guide duration of antibiotic therapy in intensive care patients: a randomized prospective controlled trial Crit Care 2009 3 R83 10.1186/cc7903 19493352 PMC2717450

[B20] Stolz D Smyrnios N Eggimann P Pargger H Thakkar N Siegemund M Marsch S Azzola A Rakic J Mueller B Tamm M Procalcitonin for reduced antibiotic exposure in ventilator-associated pneumonia: a randomised study Eur Respir J 2009 3 1364 1375 10.1183/09031936.00053209 19797133

[B21] Bouadma L Luyt CE Tubach F Cracco C Alvarez A Schwebel C Schortgen F Lasocki S Veber B Dehoux M Use of procalcitonin to reduce patients’ exposure to antibiotics in intensive care units (PRORATA trial): a multicentre randomised controlled trial Lancet 2010 3 463 474 10.1016/S0140-6736(09)61879-1 20097417

[B22] Stocker M Fontana M El Helou S Wegscheider K Berger TM Use of procalcitonin-guided decision-making to shorten antibiotic therapy in suspected neonatal early-onset sepsis: prospective randomized intervention trial Neonatology 2010 3 165 174 10.1159/000241296 19776651

[B23] Gibot S Cravoisy A Soluble form of the triggering receptor expressed on myeloid cells-1 as a marker of microbial infection Clin Med Res 2004 3 181 187 10.3121/cmr.2.3.181 15931355 PMC1069091

[B24] Gibot S Cravoisy A Levy B Bene MC Faure G Bollaert PE Soluble triggering receptor expressed on myeloid cells and the diagnosis of pneumonia N Engl J Med 2004 3 451 458 10.1056/NEJMoa031544 14749453

[B25] Bishara J Hadari N Shalita-Chesner M Samra Z Ofir O Paul M Peled N Pitlik S Molad Y Soluble triggering receptor expressed on myeloid cells-1 for distinguishing bacterial from aseptic meningitis in adults Eur J Clin Microbiol Infect Dis 2007 3 647 650 10.1007/s10096-007-0343-z 17610097

[B26] Determann RM Weisfelt M de Gans J van der Ende A Schultz MJ van de Beek D Soluble triggering receptor expressed on myeloid cells 1: a biomarker for bacterial meningitis Intensive Care Med 2006 3 1243 1247 10.1007/s00134-006-0240-4 16786330

[B27] Gamez-Diaz LY Enriquez LE Matute JD Velasquez S Gomez ID Toro F Ospina S Bedoya V Arango CM Valencia ML Diagnostic accuracy of HMGB-1, sTREM-1, and CD64 as markers of sepsis in patients recently admitted to the emergency department Acad Emerg Med 2011 3 807 815 10.1111/j.1553-2712.2011.01113.x 21762470

[B28] Suarez-Santamaria M Santolaria F Perez-Ramirez A Aleman-Valls MR Martinez-Riera A Gonzalez-Reimers E de la Vega MJ Milena A Prognostic value of inflammatory markers (notably cytokines and procalcitonin), nutritional assessment, and organ function in patients with sepsis Eur Cytokine Netw 2010 3 19 26 20146986 10.1684/ecn.2009.0185

[B29] Jeong SJ Song YG Kim CO Kim HW Ku NS Han SH Choi JY Kim JM Measurement of plasma sTREM-1 in patients with severe sepsis receiving early goal-directed therapy and evaluation of its usefulness Shock 2012 3 574 578 10.1097/SHK.0b013e318250da40 22395243

[B30] Lawn SD Myer L Bangani N Vogt M Wood R Plasma levels of soluble urokinase-type plasminogen activator receptor (suPAR) and early mortality risk among patients enrolling for antiretroviral treatment in South Africa BMC Infect Dis 2007 3 41 10.1186/1471-2334-7-41 17509133 PMC1885800

[B31] Ostrowski SR Ravn P Hoyer-Hansen G Ullum H Andersen AB Elevated levels of soluble urokinase receptor in serum from mycobacteria infected patients: still looking for a marker of treatment efficacy Scand J Infect Dis 2006 3 1028 1032 10.1080/00365540600868305 17148072

[B32] Perch M Kofoed P Fischer TK Co F Rombo L Aaby P Eugen-Olsen J Serum levels of soluble urokinase plasminogen activator receptor is associated with parasitemia in children with acute Plasmodium falciparum malaria infection Parasite Immunol 2004 3 207 211 10.1111/j.0141-9838.2004.00695.x 15491469

[B33] Koch A Tacke F Why high suPAR is not super–diagnostic, prognostic and potential pathogenic properties of a novel biomarker in the ICU Crit Care 2011 3 1020 10.1186/cc10577 22182777 PMC3388688

[B34] Donadello K Covajes C Scolletta S Taccone FS Santonocito C Brimioulle S Beumier M Vannuffelen M Gottin L Vincent JL Clinical value of suPAR, a new biomarker Intensive Care Med 2011 3 S199

[B35] Huttunen R Syrjanen J Vuento R Hurme M Huhtala H Laine J Pessi T Aittoniemi J Plasma level of soluble urokinase-type plasminogen activator receptor as a predictor of disease severity and case fatality in patients with bacteraemia: a prospective cohort study J Intern Med 2011 3 32 40 10.1111/j.1365-2796.2011.02363.x 21332843

[B36] Fine MJ Auble TE Yealy DM Hanusa BH Weissfeld LA Singer DE Coley CM Marrie TJ Kapoor WN A prediction rule to identify low-risk patients with community-acquired pneumonia N Engl J Med 1997 3 243 250 10.1056/NEJM199701233360402 8995086

[B37] Huang DT Weissfeld LA Kellum JA Yealy DM Kong L Martino M Angus DC Risk prediction with procalcitonin and clinical rules in community-acquired pneumonia Ann Emerg Med 2008 3 48 58 42 10.1016/j.annemergmed.2008.01.003 PMC2775454 18342993

[B38] Suberviola B Castellanos-Ortega A Llorca J Ortiz F Iglesias D Prieto B Prognostic value of proadrenomedullin in severe sepsis and septic shock patients with community-acquired pneumonia Swiss Med Wkly 2012 3 w13542 22430899 10.4414/smw.2012.13542

[B39] Christ-Crain M Morgenthaler NG Stolz D Muller C Bingisser R Harbarth S Tamm M Struck J Bergmann A Muller B Pro-adrenomedullin to predict severity and outcome in community-acquired pneumonia [ISRCTN04176397] Crit Care 2006 3 R96 10.1186/cc4955 16805922 PMC1550935

[B40] Huang DT Angus DC Kellum JA Pugh NA Weissfeld LA Struck J Delude RL Rosengart MR Yealy DM Midregional proadrenomedullin as a prognostic tool in community-acquired pneumonia Chest 2009 3 823 831 10.1378/chest.08-1981 19363212 PMC2818411

[B41] Yaegashi Y Shirakawa K Sato N Suzuki Y Kojika M Imai S Takahashi G Miyata M Furusako S Endo S Evaluation of a newly identified soluble CD14 subtype as a marker for sepsis J Infect Chemother 2005 3 234 238 10.1007/s10156-005-0400-4 16258819

[B42] Mussap M Noto A Fravega M Fanos V Soluble CD14 subtype presepsin (sCD14-ST) and lipopolysaccharide binding protein (LBP) in neonatal sepsis: new clinical and analytical perspectives for two old biomarkers J Matern Fetal Neonatal Med 2011 3 Suppl 2 12 14 21740312 10.3109/14767058.2011.601923

[B43] Shozushima T Takahashi G Matsumoto N Kojika M Okamura Y Endo S Usefulness of presepsin (sCD14-ST) measurements as a marker for the diagnosis and severity of sepsis that satisfied diagnostic criteria of systemic inflammatory response syndrome J Infect Chemother 2011 3 764 769 10.1007/s10156-011-0254-x 21560033

[B44] Xiao B Liu Z Li BS Tang B Li W Guo G Shi Y Wang F Wu Y Tong WD Induction of microRNA-155 during Helicobacter pylori infection and its negative regulatory role in the inflammatory response J Infect Dis 2009 3 916 925 10.1086/605443 19650740

[B45] Vigorito E Kohlhaas S Lu D Leyland R: miR-155: an ancient regulator of the immune system Immunol Rev 2013 3 146 157 10.1111/imr.12057 23550644

[B46] Vasilescu C Rossi S Shimizu M Tudor S Veronese A Ferracin M Nicoloso MS Barbarotto E Popa M Stanciulea O MicroRNA fingerprints identify miR-150 as a plasma prognostic marker in patients with sepsis PLoS One 2009 3 e7405 10.1371/journal.pone.0007405 19823581 PMC2756627

[B47] Wang H Zhang P Chen W Feng D Jia Y Xie L Serum microRNA signatures identified by Solexa sequencing predict sepsis patients’ mortality: a prospective observational study PLoS One 2012 3 e38885 10.1371/journal.pone.0038885 22719975 PMC3376145

