# Supplementary material for: Role of biomarkers in the management of antibiotic therapy: an expert panel review: I – currently available biomarkers for clinical use in acute infections
Source: Ann Intensive Care. 2013 Jul 9;3:22. doi: 10.1186/2110-5820-3-22 (PMC3708786; doi:10.1186/2110-5820-3-22)
Supplement: Additional file 1: Table S1 — List of biomarkers tested in the field of infectious diseases. [file 2110-5820-3-22-S1.doc]

**On-line Supplement Table to Part I:**

**Role of biomarkers in the management of antibiotic therapy: An expert panel review**

**I – Currently available biomarkers for clinical use in acute infections**

Anne-Marie Dupuy, François Philippart, Yves Péan, Sigismond Lasocki, Jean-Pierre Quenot, Nicolas Roche, Pierre-Emmanuel Charles, Yann-Eric Claessens, Martin Chalumeau, Stéphanie Ruiz, Christele Gras-Le Guen, Charles-Edouard Luyt, Jean-Pierre Bedos, Jérôme Pugin, Jean-Paul Stahl, Rémy Gauzit, Benoit Misset, Christian Brun-Buisson, for the Maurice Rapin Institute Biomarkers Group.

**The following Table lists biomarkers tested in the field of infectious diseases:**

**Sa. in adults**

**Sb. in children**

**Sc. in neonates**

**Sa. Biomarker tested in adults**

| **Biomarkers** |  |  | |  | |  | |  |  |  |  |
| --- | --- | --- | --- | --- | --- | --- | --- | --- | --- | --- | --- |
| **Cell type** | Polymorphonuclears (PMN) | Monocytes (MC) | | PMN & MC | | Eosinophils | | Neutrophils | Soluble receptors |  |  |
| **Cell surface marker / soluble receptors** | CD11b/CD11c/  CD66b/  TLR2/TLR4  ROS | CD11c/CD14/  TLR2/  TLR4  & CD14s  ROS | | HLA-DR CD11b/ICAM1(CD54)/  CD66b/CD63/CD64 | | CRTH2 CCR3 | | nCD64 | sCD14 sCD14-ST sE-selectin  sFlt-1 (sVEGFR1) sELAM-1 sTREM-1 suPAr |  |  |
|  |  |  |  | |  | |  | |  |  |  |
| **cytokines** | All | TNF IL-6 IL-8 IL-10 IL-1ra IL-1b | | VEGF sVEGFR1 | | MIF uMIF uMIF/Cr | | HMGB-1 | IL-4 MCP-1 G-CSF | IP-10 |  |
|  |  |  | |  | |  | |  |  |  |  |
| **Acute phase proteins** | CRP | LBP | | LPS | | Ferritin | | SAA | Pentraxin 3 |  |  |
| **Pro-hormones** | PCT | ProADM | | proANP | | MR-ProADM | |  |  |  |  |
| **Coagulation markers** |  | aPTT waveform analysis | | ICAM-1 VCAM-1 PAI-1 | | thrombomodulin | |  |  |  |  |
| **others** | Lactates | Differential count of immature PMN (DNI) | | Leucocytes | | Neopterin (blood and urine) | | sPLA2 | Elastase | C3a | Endotoxin Lactoferrin |

**Sb**. Biomarkers tested in children

| **Biomarkers** |  |  |  |  |
| --- | --- | --- | --- | --- |
|  | PMN | Soluble |  |  |
| **Surface receptors and soluble receptors** | CD64 | sCD14-ST (or presepsin) |  |  |
|  |  |  |  |  |
| **cytokines** | MIF uMIF uMIF/Cr |  |  |  |
|  |  |  |  |  |
| **Acute phase proteins** | CRP | LBP | LPS | Ferritin |
| **Pro-hormones** | PCT |  |  |  |
| **Coagulation markers** | aPTT waveform analysis |  |  |  |
| **Others** | Lactates |  |  |  |

**Sc**. Biomarkers tested in neonates

| **Biomarker** |  |  |  |  |  |
| --- | --- | --- | --- | --- | --- |
|  | PMN | Soluble |  |  |  |
| **Cell surface marker and soluble receptors** | CD64 | sCD14-ST ( presepsin) |  |  |  |
|  |  |  |  |  |  |
| **cytokines** | MIF uMIF uMIF/Cr | IL6, TNF, IL8, G-CSF, IP-10 |  |  |  |
|  |  |  |  |  |  |
| **Acute phase proteins** | CRP | LBP | LPS | Ferritin | SAA |
| **Pro-hormones** | PCT |  |  |  |  |
| **Coagulation markers** | aPTT waveform analysis |  |  |  |  |
| **others** |  | Differential count of immature PMN (DNI) | sPLA2 |  |  |

**Abbreviations:**

ADM and pro-ADM: Adrenomodullin and pro-adrenomodullin; aPTT : activated Partial Thromboplastin Time; CCR3: Chemokine (C-C motif) receptor 3; CRP: C-Reactive protein; CRTH2: Chemoattractant Receptor-Homologous molecule expressed on Th2; CSF: DNI: Differential count of immature PNN; G-CSF: Granulocyte colony-stimulating factor; HLA: Human Leukocyte Antigens; HMGB1: High Mobility Group protein B1; ICAM 1: Intercellular Adhesion Molecule 1; IFN-ϒ: Interferon-gamma; IL: Interleukine; IP-10: Interferon gamma-induced Protein 10; LBP: Lipopolysaccharide binding protein; LPS: Lipopolysaccharide; MC: monocytes; MCP-1: Monocyte chemotactic protein-1; MIF: Macrophage migration Inhibitory Factor; MR-proADM: mid-region pro-adrenomedullin; PAI 1: Plasminogen Activator Inhibitor 1; PCT: Procalcitonin; PMN: Polymorphonuclear neutrophil; ProADM: proAdrenoMedullin; ProANP: proAtrial natriuretic peptide; ROS: Reactive oxygen species; SAA: Serum Amyloid A protein; sCD14-ST: Soluble CD14 SubType; sELAM : soluble endothelial leucocyte adhesion molecule-1; sFlt-1: Soluble fms-like tyrosine kinase-1 or sVEGFR1; sPLA2: soluble Phospholipase A2; sTREM-1: soluble Triggering Receptor Expressed on Myeloid cells-1; SuPAr: Soluble urokinase-type Plasminogen Activator receptor; sVEGFR1: Vascular endothelial growth factor receptor 1 soluble; TNF: Tumor necrosis factor; TLR-2 or 4: Toll-like receptor 2 or 4; uMIF: Urinary Macrophage migration Inhibitory Factor; uMIF/cr : uMIF/creatinine: VCAM-1: Vascular Cell Adhesion Molecule 1; VEGF: vascular endothelial growth factor
